# The Effects of Serum and Follicular Fluid Vitamin D Levels on Assisted Reproductive Techniques: A Prospective Cohort Study

**DOI:** 10.22074/IJFS.2021.138605.1033

**Published:** 2021-10-16

**Authors:** Ghazal Neysanian, Mahboube Taebi, Atefeh Rezaeian, Mohammad Hossein Nasr-Esfahani, Maryam Jahangirifar

**Affiliations:** 1. Department of Midwifery and Reproductive Health, Isfahan University of Medical Sciences, Isfahan, Iran; 2. Department of Animal Biotechnology, Reproductive Biomedicine Research Center, Royan Institute for Biotechnology, ACECR, Isfahan, Iran; 3. Isfahan Fertility and Infertility Center, Isfahan, Iran; 4. School of Nursing and Midwifery, Faculty of Medicine, Nursing and Health Sciences, Monash University, Melbourne, Australia

**Keywords:** Assisted Reproductive Techniques, Follicular Fluid, Infertility, Serum, Vitamin D

## Abstract

**Background:**

Based on studies on animal models, vitamin D plays an essential role in reproduction by controlling Ca and Mg levels. Despite these findings, the effects of vitamin D deficiency and supplementation on the outcome of assisted reproductive techniques (ART) remain controversial. Therefore, the aim of the present study was to assess the relationship between serum and follicular fluid 25-OH vitamin D levels on reproductive outcomes of infertile women.

**Materials and Methods:**

This prospective cohort study included 150 infertile women who underwent *in vitro* fertilization (IVF) or intracytoplasmic sperm injection (ICSI). The participants were allocated to one of the three groups according to their serum and follicular fluid 25-OH vitamin D concentrations (less than 10 ng/ml, between 10 and 30 ng/ ml and more than 30 ng/ml), and fertilization, cleavage and biochemical and clinical pregnancy rates were compared among the groups. Data was analyzed by SPSS software and using Chi-square and Spearman correlation coefficient.

**Results:**

Serum and follicular fluid vitamin D levels significantly correlated with biochemical (P=0.008), (P=0.003) and clinical pregnancy (P=0.017), (P=0.001) rates respectively . However, the quality of embryos (P=0.125), (P=0.106) and fertilization rate (P=0.082), (P=0.059) were not associated with the level of serum and follicular fluid vitamin D.

**Conclusion:**

This study found that women with higher levels of vitamin D in their serum and follicular fluid are significantly more likely to achieve pregnancy but without affecting the quality of embryo and fertility rate.

## Introduction

Infertility is a widespread problem, which affects many humans in the world. Today approximately 20% of couples are facing this problem. While more than half of them are seeking treatment options, approximately one-quarter of them accept child absenteeism (1). Generally, infertility is defined as the inability of couples to get pregnant after one year of regular unprotected sexual intercourse (2). It could be the result of a disease, stressful lifestyle, consumption of unhealthy foods and chemical medicines and exposure to industrial and environmental pollutants or other reasons (3). The most effective and costeffective way to prevent fertility problems or to treat these issues is nutritional modifications. Different food supplementations have significant roles in both prevention and treatment of infertility by their impact on the female and male reproductive systems. For instance, a deficiency in some vitamins and minerals can lead to infertility, and in fact, there have been recent reports suggesting a role for vitamin D in infertility (4)

The role of vitamin D in biological processes such as cell growth, metabolism modification, especially insulin function, autoimmune system and cardiovascular health is well known (5). This vitamin plays its role by interacting with vitamin D receptors (VDR) on various organs in the body (6). The presence of VDR in reproductive tissues such as testis, placenta, uterus and ovary has lead to the possibility that this vitamin is involved in reproductive processes as well (7). Disorders such as reduced fertility, diminished mating success, increased pregnancy complications, gonadal insufficiency, hypogonadism, uterine hypoplasia, impaired folliculogenesis (8) and infertility caused by vitamin D deficiency have been reported in animal models and human (9). There are several studies, which support the role of this vitamin in calcium transport through the placenta (10), placental steroidogenesis (11) and decidualization of endometrium (12). In addition, its function as a regulator of key target genes, which are related to implantation and establishment of the fetoplacental unit (13) has been identified.

It is believed that the vitamin D level in follicular fluid can be associated with its level in the body resources (14). There are contradictory results regarding the impact of this vitamin on the number of oocytes (8, 14) and embryo quality (15, 16) in assisted reproduction technique (ART). Follicular fluid, derived from both the follicular cell secretions and plasma (17), can be an important indicator of vitamin D levels, as it has been shown that serum vitamin D levels are related to the amount of this vitamin in the follicular fluid (15). 

The presence of follicular fluid in many species shows its potential role in ovarian physiology, steroidogenesis (18), follicular growth and ovulation, oocyte maturation (19), and their transmission to fallopian tube (17). As follicular fluid provides a suitable environment for optimal growth of oocytes, which can have a direct effect on fertility (20), this study aimed to investigate the effects of serum and follicular levels of vitamin D on fertility and ART outcomes.

## Materials and Methods

A prospective cohort study was performed on 150 women aged 18-40 years old with primary infertility, who had undergone assisted reproductive treatments [intracytoplasmic sperm injection + *in vitro* fertilization (ICSI+IVF)] at Isfahan Fertility and Infertility Center, Isfahan, Iran, from April to September 2015. A simple sampling design was used. Women who met the inclusion criteria were included in the study. 

The inclusion criteria for this study consisted of female infertility, lack of endocrine disorders such as Cushing’s syndrome, Hyper or Hypothyroidism, hyperprolactinemia (8), body mass index (BMI) 18-29 kg/m^2^ (20), lack of congenital uterine anomalies and endometriosis (20), and not consuming drugs affecting vitamin D metabolism (21). The following formula was used for calculating the sample size:


n=z2.s2d2
z = 1. 96s = an estimate of the standard deviation of vitamin D leveld= accuracy that considered 0.16.

After receiving the standard long gonadotropin-releasing hormone (GnRH)-a protocol by all the subjects, Buserelin Acetate 0.5 mg/day was injected intramuscularly on day 20-21 of menstrual cycle and 0.25 mg/day after mensuration until ovum pick up day. Then a subcutaneous injection of 75 IU/day recombinant folliclestimulating hormone (FSH) was administered for ovarian stimulation. When at least two follicles reached 18-2 mm, human chorionic gonadotropin (HCG) 10,000 IU was administered through an intramuscular injection. After 34-36 hours, ovum was picked up. All the participants were followed by sequential vaginal ultrasound.

**Fig.1:**
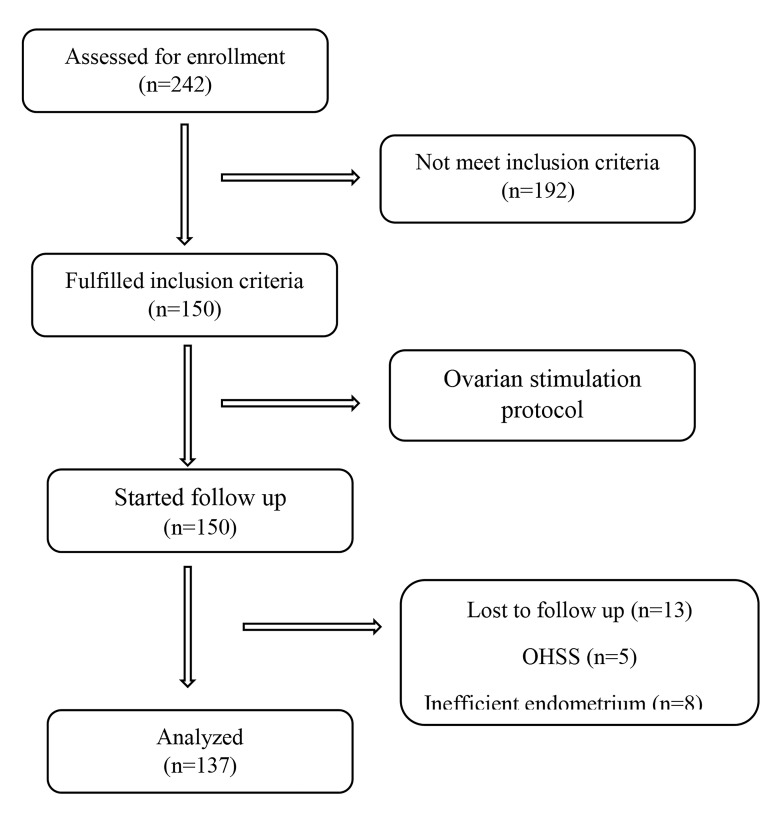
Study flowchart. OHSS; Ovarian hyperstimulation syndrome.

On the same day of ovum pick up, follicular fluid and serum samples were collected to determine the level of 25-OH vitamin D (HPLC system, euro immune kit, Gerrmany). Vitamin D levels were defined as sufficient (30- 100 ng/ml), insufficient (10-30 ng/ml), or deficient (<10 ng/ml). Fertilization was performed in the lab by an expert embryologist and the success rate of fertilization and embryo quality were investigated according to the number of blastomeres and fragmentation rate. Good-quality embryos (7 or more blastomeres and >20% fragmentation rate) were transferred to uterine 3 days after fertilization. Pregnancy was detected by serum β-hCG analysis (Electrochemiluminescence method, Roche, Germany) two weeks after embryo transfer, and a transvaginal ultrasound scan was employed at 3-4 weeks later to detect the intrauterine gestational sac. Itn this study, after ovum pick ups in all the women, luteal phase was supported with 400 mg suppository vaginal progesterone (cyclogest) twice per day for 10-12 weeks after pregnancy (Fig .1).

### Ethical considerations

This study was approved by Ethics Committee of Isfahan University of Medical Sciences, Isfahan, Iran (IR. MUI.REC.1394.3.147). A written informed consent was taken from each of the participants of this research.

### Data analysis

Data were analyzed by SPSS software (version 16, SPSS Inc., Chicago, Ill., USA) and Chi-square and Spearman correlation coefficient. A value of P≤0.05 was considered statistically significant.

## Results

This study included 150 infertile women. Thirteen of them were excluded from the study due to ovarian hyperstimulation syndrome (OHSS) or because their endometrium was not ready for emberyo transfer. Ultimately, data analysis was performed on 137 patients. The patients’ demographic characteristics are summarized in Table 1. The age of the females ranged from 21-40 with an average age of 30.30 ± 4.75 years. The mean of vitamin D level of follicular fluid and the serum was 26.99 ± 24.32 ng/ml and 26.37 ± 24.36 ng/ ml, respectively. Also, there was a significant difference between the levels of vitamin D in serum and follicular fluid (r=0.711, P<0.001).

The results show that 45.8% of the subjects with positive biochemical pregnancy had serum vitamin D levels above 30 ng/ml, while 80.5% of the women with negative pregnancy rate were in the group with vitamin D levels less than 10 ng/ml. The statistical analysis indicated that there was a positive association between serum vitamin D levels and the incidence of biochemical pregnancy (P=0.008) and clinical pregnancy (P=0.017, Table 2).

In addition, in 44.4% of the females who had a positive biochemical pregnancy, the vitamin D level in follicular fluid was higher than 30 ng/ml, while in 84.6% of the women who had less than 10ng/ml vitamin D in their follicular fluid, a successful pregnancy did not occur. Statistically, a positive association was found between follicular fluid vitamin D levels and pregnancy (biochemical pregnancy, P=0.003, chemical pregnancy, P=0.001) (Table 2).

**Table 1:** Baseline characteristics of study population (n=137)


Characteristics		Mean ± SD or n (%)

Woman age (Y)		30.30 ± 4.75
Education level		
	Graduate	69 (50.3)
	High school	42 (30.6)
	Middle school	15 (10.9)
	Elementary	11 (8.2)
Occupation		
	Housewife	104 (75.9)
	Employed	33 (24.1)
Location		
	Urban	128 (93.4)
	Rural	9 (6.6)
Duration of infertility (Y)		6.16 ± 4.49
BMI (Kg/m^2^)		24.70 ± 2.87
Follicular fluid vitamin D (ng/ml)		26.99 ± 24.32
Serum level of vitamin D (ng/ml)		26.37 ± 24.36


BMI; Body mass index.

Table 3 shows that there is no association between the serum level of vitamin D and the quality of embryo (r=0.126, P=0.125) or fertilization rate (r=0.019, P=0.082). Similarly, no associations were observed between the follicular fluid level of vitamin D and embryo quality (r=0.133, P=0.106) or fertilization rate (r=0.154, P=0.059). 

**Table 2:** Serum and follicular fluid vitamin D levels and biochemical and clinical pregnancy rate


		Follicular fluid vitamin D (ng/ml)			P value^*^	X^2^	Serum vitamin D (ng/ml)			P value^*^	X^2^
		10>	10-30	30≤			10>	10-30	30≤		

Biochemical pregnancy											
	Positive	6 (15.4)	14 (31.8)	24 (44.4)	0.003	8.661	8 (19.5)	14 (29.2)	22 (45.8)	0.008	7.094
	Negative	33 (84.6)	30 (68.2)	30 (55.6)			33 (80.5)	34 (70.8)	26 (54.2)		
Clinical pregnancy											
	Positive	3 (7.7)	13 (29.5)	22 (40.7)	0.001	11.938	6 (14.6)	14 (29.2)	18 (37.5)	0.017	5.653
	Negative	36 (92.3)	31 (70.5)	32 (59.3)			35 (85.4)	34 (70.8)	30 (62.5)		


Data are presented as n (%). *; P≤0.05 was considered significant.

**Table 3:** Follicular fluid vitamin D levels and embryo quality and fertility rate


		10>	10-30	30≤	P value*

Follicular fluid vitamin D (ng/ml)					
	Embryo quality	59.01 ± 28.72	66.45 ± 32.22	68.33 ± 28.23	0.106
	Fertilization rate	55.12 ± 30.63	57.96 ± 30.94	66.62 ± 23.87	0.059
Serum vitamin D (ng/ml)					
	Embryo quality	60.79 ± 30.29	64.97 ± 29.16	68.72 ± 30.23	0.125
	Fertilization rate	60.65 ± 30.24	57.98 ± 29.30	62.40 ± 26.95	0.082


Data are presented as mean ± SD. *; P≤0.05 was considered significant.

## Discussion

In this study, we sought to elucidate one of the most controversial issues in fertility, which is whether vitamin D affects assisted reproduction outcomes. Over the past decades there has been extensive investigation on the physiological roles of 25-OH vitamin D on ART outcomes, but the results of previous studies are very heterogeneous (22). This heretogenousity can be due to various factors affecting vitamine D levels including diet and the degree of exposure with sunlight (23). Nonetheless, independently of these factors, animal experimental studies have shown that vitamin D deficiency may affect fertility through Ca dependent/independent hemostasis (24). However, most reproductive consequnences of viatmin D deficiency are corrected. Consequently, this prospective cohort study was performed to evaluate the association of vitamin D levels in follicular fluid and serum on both biochemical and clinical pregnancy outcomes. We also evaluated the association between these two parameters with embryo quality and fertilization rates among participants. 

It is noteworthy that the relationship between the level of 25-OH vitamin D in follicular fluid is a reflective of stores of vitamin D in the body (8, 14). Moreover, serum and follicular fluid 25(OH)D are directly related to each other (25).

Our results showed a significant association between the serum and follicular fluid vitamin D levels and pregnancy rate, which is in agreement with several previous published work (4, 8, 16, 26, 27), however, it is in contrast to other studies, which have reported no association (14, 25, 28- 30) or inverse relation (15). 

We also concluded that Vitamin D status is not associate with embryo quality and fertilization rates, despite the fact that association ofthe fertilization rate was close to be significant. These results are in accordance with Rudick et al. (16) and Aleyasin et al. (31). While Anifandis et al. (15) found that higher levels of this vitamin have a negative impact on embryo quality and therefore on IVF outcome. 

Rudick et al. (16) showed an association between vitamin D and IVF success rate among non-Hispanic whites but not in Asians. They concluded that there was a statistically significant impact of race on the relationship between these two parameters. Our current finding are in contrast with previous results of investigations in Iran (14, 29) that suggested no relationship between vitamin D levels and the outcomes of ART. Definitely, the most surprising results were observed by the Anifandis et al. (15), as they reported an excess level of vitamin D in combination with a decreased level of follicular fluid glucose have an adverse effect on ART outcomes of infertile Greek women. 

Considering the substantial discrepancies with published works, these results add to the literature the potential role of vitamin D on pregnancy rate among infertile couples undergoing infertility treatments. The possible mechanism can be explained as follows: firstly, vitamin D has been diagnosed as a factor, which affects endometrium receptivity. 1,25-dihydroxy vitamin D_3_ (1,25[OH]_2_ D_3_) is produced in endometriotic cells in response to interleukin B1, which is secreted by blastocyst. This enzyme binds to VDRs on the endometrium and regulates the expression of genes involved in implantation and placental development (32). Additionally, vitamin D plays a critical role in upregulation of transcription of *HOXA10* gene, an important gene participating in both placentation and implantation (33). It is important to point out that *HOXA10* gene can be activated by interaction with vitamin D (34).

Secondly, the influence of vitamin D on development of follicles and embryo has been previously reported (23). This vitamin also stimulates the production of estradiol, estrone, and progesterone and the enzymes that are responsible for the production of these hormones have vitamin D response element in their promotors (24). Additionally, anti mullerian hormone (AMH), a marker of ovarian reserve, has an inhibitory effect on the primordial follicle recruitment during folliculogenesis. It has been shown that there is a functional VDR element (VDRE) in the promoter of *AMH* gene (24) and that AMH is positively affected by vitamin D. Therefore, defects in VDR or its deficiency in the body can retard follicle development and oocyte maturation (14). Thus, it is not surprising to see some reports regarding the relationship between level of vitamine D and low ovarian response, as well as the fact that viatmine D supplementation increases AMH level (24).

Finally, vitamin D plays a vital role in gestation and maintaining a healthy pregnancy. The association between a decreased level of vitamin D and a higher risk of gestational diabetes and preeclampsia has been investigated by several studies (35).

Considering all the above mentioned findings, it is not unexpected to observe that vitamin D plays a crucial role in fertility outcomes. Therefore, the discrepancy within published works can be explained by other confounding factors, such as a source of vitamin D (diet, exposure to the sun, former supplementation), lifestyle, ethnicity, age, BMI, seasonal effect, and involvement of other ovarian factors responsible for this procedure.

In this research certain limitations should be considered; although we investigated maternal vitamin D status, the paternal vitamin D concentration needs to be assessed simultaneously. There are numerous studies showing that deficiency in vitamin D not only effects sperm parameters but also affects sperm DNA integrity, which subsequently will affect embryo developmental competency (35). Additionally, there is a lack of monitoring and measurement of vitamin D levels during pregnancy until delivery. We analyzed our data in terms of biochemical and clinical pregnancy to help better understand these issues. And finally, although we provided sufficient results through this study, it is nearly impossible to measure all the various confounding factors.

Indeed, a possible association has been reported among vitamin D and small for gestational age (SGA) infants (37- 39), preeclampsia (35), and gestational diabetes mellitus (GDM) (40). Therefore, the side effect of vitamin D supplementation during pregnancy should be considered before suggesting its widespread consumption.

## Conclusion

The findings of this study revealed that there is a positive association between serum and follicular fluid vitamin D levels and the success rate of biochemical and clinical pregnancy. However, there was no significant relationship between vitamin D level of follicular fluid and embryo quality or fertilization rate. Vitamin D supplementation is suggested to increase the level of this vitamin to a normal range in women with an insufficient level of vitamin D for achieving successful biochemical and clinical pregnancy.
